# The paediatrician workforce and its role in addressing neonatal, child and adolescent healthcare in Kenya

**DOI:** 10.1136/archdischild-2019-318434

**Published:** 2020-06-18

**Authors:** Mike English, Brigid Strachan, Fabian Esamai, Thomas Ngwiri, Osman Warfa, Patrick Mburugu, Grace Nalwa, Jesse Gitaka, John Ngugi, Yingxi Zhao, Paul Ouma, Fred Were

**Affiliations:** 1 KEMRI-Wellcome Trust Research Programme Nairobi, Nairobi, Kenya; 2 Oxford Centre for Global Health Research, Nuffield Department of Medicine, University of Oxford, Oxford, Oxfordshire, UK; 3 Independent Consultant, Oxford, UK; 4 Department of Paediatrics and Child Health, College of Health Sciences, Moi University, Kenya, Eldoret, Kenya; 5 Kenya Paediatric Association, Nairobi, Kenya; 6 Ministry of Health, Nairobi, Kenya; 7 Department of Paediatrics and Child Health, School of Medicine, Jomo Kenyatta University of Africulture and Technology, Nairobi, Kenya; 8 Department of Paediatrics and Child Health, Maseno University, Maseno, Nyanza, Kenya; 9 Directorate of Research and Innovation, School of Medicine, Mount Kenya University, Thika, Kenya; 10 Department of Paediatrics and Child Health, Kenyatta University, Nairobi, Kenya; 11 Department of Paediatrics and Child Health, University of Nairobi, Nairobi, Kenya

**Keywords:** general paediatrics, health services research, medical education, paediatric practice, tropical paediatrics

## Abstract

**Objective:**

To examine the availability of paediatricians in Kenya and plans for their development.

**Design:**

Review of policies and data from multiple sources combined with local expert insight.

**Setting:**

Kenya with a focus on the public, non-tertiary care sector as an example of a low-income and middle-income country aiming to improve the survival and long-term health of newborns, children and adolescents.

**Results:**

There are 305 practising paediatricians, 1.33 per 100 000 individuals of the population aged <19 years which in total numbers approximately 25 million. Only 94 are in public sector, non-tertiary county hospitals. There is either no paediatrician at all or only one paediatrician in 21/47 Kenyan counties that are home to over a quarter of a million under 19 years of age. Government policy is to achieve employment of 1416 paediatricians in the public sector by 2030, however this remains aspirational as there is no comprehensive training or financing plan to reach this target and health workforce recruitment, financing and management is now devolved to 47 counties. The vast majority of paediatric care is therefore provided by non-specialist healthcare workers.

**Discussion:**

The scale of the paediatric workforce challenge seriously undermines the ability of the Kenyan health system to deliver on the emerging survive, thrive and transform agenda that encompasses more complex health needs. Addressing this challenge may require innovative workforce solutions such as task-sharing, these may in turn require the role of paediatricians to be redefined. Professional paediatric communities in countries like Kenya could play a leadership role in developing such solutions.

**What is already known on this topic?**

- Kenya and many low and middle income countries face critical shortages and maldistribution of all health workers which undermines access to and quality of care.
- Countries like Kenya aspire to deliver on a universal health coverage agenda and address broader neonatal, child and adolescent health needs. Meeting all these needs will require an appropriately skilled workforce.
- There are few reports that focus on the development of the paediatric workforce.

**What this study adds?**

- We provide a careful description and analysis of the paediatric workforce and its distribution in Kenya.
- Data suggest large parts of the country have none or only one paediatrician in the public sector, that there are few other cadres with specific training in neonatal, child or adolescent health and that the current production of doctors in general and paediatricians in particular remains very limited.
- To meet future needs of new borns, children and adolescents, the roles and training of paediatricians should be reviewed alongside other workforce innovations to enable equitable access to quality care.

## Introduction

The global health workforce crisis is particularly acute in African countries^1^ and the child health workforce is a key priority of WHO and the United Nations.^2 3^ They advocated in the Every Women Every Child: Global Strategy for Women’s, Children’s and Adolescents’ Health 2016–2030, for adequate financing, workforce development and monitoring to assist governments to expand equitable coverage.^4 5^ This requires the development of a competent and motivated workforce that is designed for the local context.^3^ As part of this, attention must be paid to the number and roles of specialists.^6^ Here, we reflect on progress in developing the paediatrician workforce in Kenya 15 years after raising initial concerns about the absence of broad strategic thinking in this area.^7^ Others have recently described the global distribution of paediatricians and the 140-fold difference in number per 100 000 population in a report placing Kenya at a similar level of development to many low-income and middle-income countries (LMICs).^8^

Kenya’s population is estimated at approximately 51 million and approximately 50% are part of the paediatric population defined as from birth to 18 years in Kenyan policy.^9^ By 2050, the Kenyan population is projected to nearly double. This youthful population presents significant challenges in the immediate and long term to health service planning, for meeting the Sustainable Development Goals (SDG) and delivering on the Universal Health Coverage (UHC) agenda.^10^ After constitutional changes in Kenya in 2013, the national government became responsible for policy, planning and regulation for health and, of relevance to this report, for planning and regulation of medical education at undergraduate and postgraduate levels. Wider stakeholders in these strategic and operational considerations include universities, training institutions, professional bodies and regulatory boards. Forty-seven county governments are responsible for local health systems. This includes local strategic workforce planning, recruitment and promotions, performance management and payment of salaries from budgets allocated to counties by the central Treasury and enhanced through local revenue generation. Here, we describe the current status and production of the paediatrician workforce in Kenya with a specific focus on the public sector that predominates in provision of specialist medical services for rural and poor urban families who form the majority of the population.

## Methods

We began by examining the policies and strategies that are intended to guide health workforce development in Kenya and prior reports assessing the availability of human resources for health and the outputs of medical schools. To achieve this, we first identified all relevant government websites and hand-searched them for published and draft reports that are publicly accessible. Second, we used a snowballing approach to identify additional documents or reports. This started with asking the authors, who are from institutions including the paediatric professional association, government departments and medical schools, for any additional official reports they could identify. We then proceeded to ask further contacts of the authors for reports until we were satisfied no major, important source document relevant to our specific question had been overlooked. Reviewed documents are either formally referenced or listed in online supplementary material.

10.1136/archdischild-2019-318434.supp1 Supplementary data

We then used these reports to describe: i) the existing paediatric and non-specialist medical graduate workforce at national level, ii) the production of these cadres in Kenya and more specifically iii) the distribution of paediatricians in county hospitals which is where such specialists are largely based within the public sector. In a second strategy, to address missing information or cross-check initial findings, the authors requested specific information from relevant institutions. These included the Kenya Paediatric Association, who maintain a register of all paediatricians who are members, the Kenya Medical and Dental Practitioners’ Board, who maintain a database of medical practitioners with a license to practice and university contacts to provide information on paediatricians in training. The authors also contacted counties’ child health focal persons to make direct enquiries about the current public sector employment of paediatricians in county hospitals in March–April 2019 and were provided with a similar summary of data from a national Health Facility Assessment exercise conducted in 2018.^11^ To try and ensure our situation analysis is as accurate as possible, we triangulated the different sources of data and as results were developed the authors reflected on whether the emerging findings were consistent with their knowledge and experience.

## Results

### Medical workforce aspirations

Kenyan Ministry of Health goals were elaborated in the Health Sector Human Resources Strategy 2014–2018 (HR Strategy 2014–2018)^12^ and more recently the Kenya Human Resources for Health Strategic Plan 2019–2023 (final draft).^13^ This strategy aimed to ensure that the government sector provides: an adequate supply and equitably distributed health workforce, a conducive environment that attracts and retains health workforce and improved human resource planning and development.^12^ The norms were developed using the WHO, Workload Indicators of Staffing Need methodology^14^ and focused on the workload components within the Kenya Essential Health Package 2014. Activity standards for staff cadres are based on local and international expert opinion. Workforce needs were further informed by policy suggesting that first referral level hospitals serving populations of at least 100 000 (level 4 facilities in Kenya) should have two paediatricians and one neonatologist while secondary referral hospitals serving populations of approximately 1 million people (level 5 facilities in Kenya) should have four paediatricians, one paediatric endocrinologist, one paediatric nephrologist, one paediatric neurologist, one paediatric surgeon and two neonatologists.^15^

Based on the HR Strategy 2014–2018, a target was set to increase the total medical physician workforce (specialist and non-specialist doctors) in the public sector in Kenya from a reported 5660 in 2015^16^ to 19 255, by 2030.^15 17^ The total medical physician workforce gap based on this target was 13 955 in 2015. There is no single regularly updated source document that enumerates health worker employment and distribution in Kenya. Using data from current medical physician registrations, we estimate there were a total of 7751 practitioners in 2019, of whom only 4116 are listed as currently actively employed (unpublished data, Kenya Medical and Dental Practitioners’ Board, July 2019). Against the 2030 target, the total public sector medical physician workforce gap in 2019 was therefore 15 139 qualified physicians.

### Medical school graduates and specialty training in paediatrics and child health

**Figure 1:**
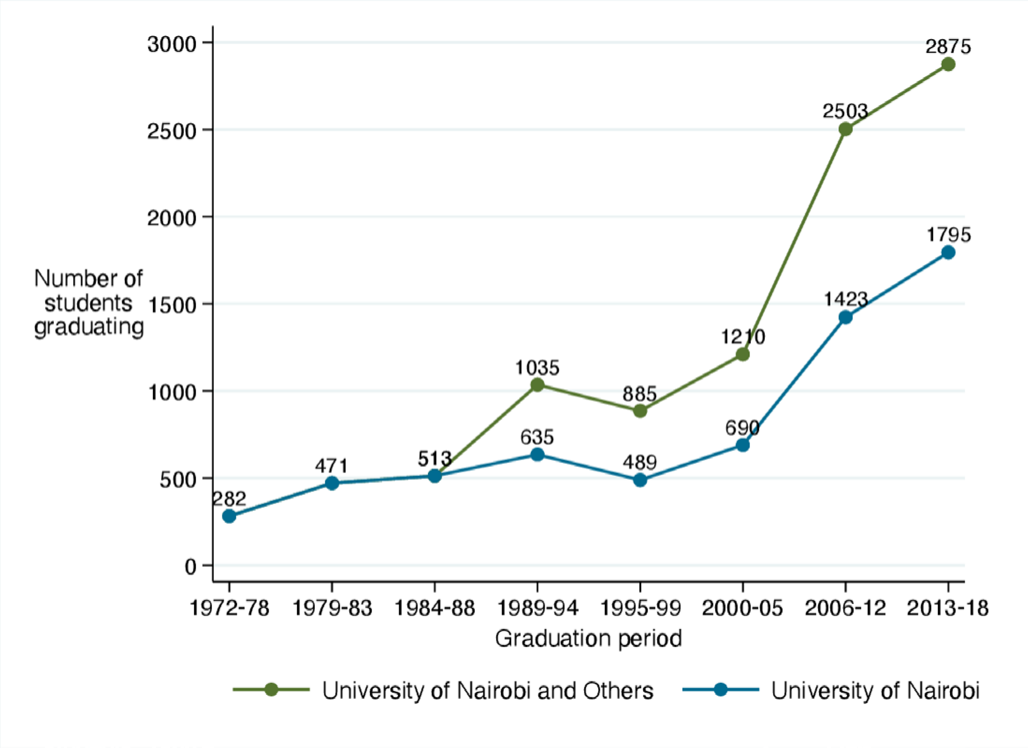
Growth in the number of medical graduates between 1972 and 2018 aggregated within 5-year period as the two earliest established medical schools were joined by new medical schools from 2004. Source: Kenya Medical Practitioners and Dentists Board, 2019.

Medical schools in Kenya have grown from 2 in 2004 to 12 in 2019.^18^ As a result, graduate output of medical physicians continues to grow and reached approximately 500–600 per year by 2018 (figure 1).^13^ Based on this growth, approximately 9000 new graduate medical physicians could be produced by 2030, potentially narrowing the gap between Kenya’s workforce aspirations and existing numbers. This simple projection however, ignores key workforce issues such as retention and complete exits from the profession or from the public sector to the private or not-for-profit sectors. It is estimated currently that only 60% of non-specialist physicians are employed in the public sector^12^ while in Kenya’s largest medical school the majority of undergraduate medical students are privately funded perhaps making them less likely to pursue public sector careers.

Typically trainees in paediatrics in Kenya are expected to have gained at least 2 years’ experience as a licensed medical practitioner prior to postgraduate training. Trainees register with one of three universities and do a 3 or 4 years Master’s in Medicine, Paediatrics and Child Health degree while undertaking supervised clinical practice in major national or university affiliated hospitals. The annual output of these provisionally qualified general paediatricians in Kenya is about 30 a year. However, our experience is that over 60% of paediatric trainees are privately funded and so only a minority enter employment in public sector county hospitals annually.

### Paediatrician numbers

Paediatricians provide neonatal, child and adolescent health services and the HR Strategy 2014–2018 recommended that by 2030, to cover all these areas, Kenya should employ 1416 in public hospitals. According to the records of the Kenya Paediatric Association, in 2019 there are a total of 601 registered paediatricians. However, this includes those across all sectors, those who are retired and in administrative and university teaching positions. In fact, only 365 paediatricians were registered with the country’s medical board in 2019 as licensed to practice.

Data from the Health Facility Assessment Survey conducted in 2018 indicated the total number of paediatricians in all hospitals in all sectors and including the public tertiary referral hospitals was 305.^11^ This survey did not include stand-alone private clinics that provide only outpatient care. It suggested over 50% of all hospital paediatricians identified were located in the cities of Nairobi and Mombasa. Furthermore, this survey’s findings indicated the total number of paediatricians in public sector county hospitals was 94. Data provided by the Child Health Focal Persons from the 47 counties in early 2019 (personal communication, 2019) suggest there were 95 paediatricians in public county hospitals (both level 4 and level 5 facilities) helping to confirm the likely accuracy of this figure.

**Figure 2:**
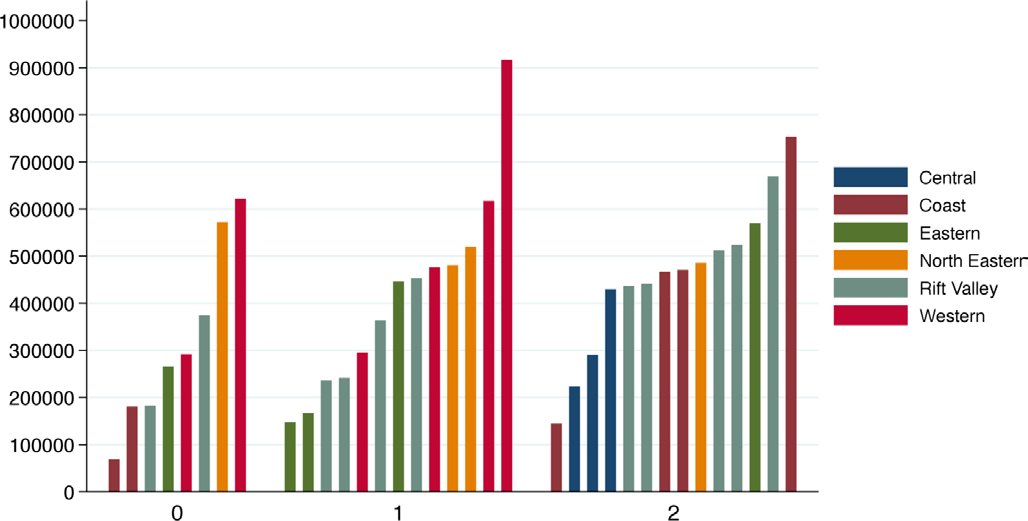
Population of those aged 18 years and younger (Y-axis) in counties with zero (n=8), one (n=13) or two (n=14) paediatricians in public sector hospitals based on data obtained in early 2019. The region of Kenya in which counties are located is indicated by the colour of the bar and explained in the legend. Additional paediatricians may be found in private or faith-based facilities in the counties represented in this figure but counties (n=12) with more than two public sector paediatricians or counties with a clinical medical school which would result in their being additional paediatricians have been excluded.

If the 305 paediatricians licensed to practice are included then Kenya has 1.33 paediatricians per 100 000 of the population aged under 19 years, just above the median for Africa and South East Asia.^8^ If we consider only public sector paediatricians in county hospitals, and exclude those in tertiary hospitals and medical school staff, the ratio falls to 0.41. Expressed differently, there is on average one paediatrician providing non-tertiary care for a quarter of a million under 19 year of age. In 21/47 counties, there was either no or one paediatrician, even in some counties with an under 19 years of age population of nearly 1 million (figure 2, data from county child health focal persons, 2019). This means for large geographic areas Kenya has similar numbers of paediatricians to the lowest-income countries in the world.

#### Financing the future workforce

In our efforts to identify policy and strategy documents guiding workforce development, we did not find any financial or budgetary projections to estimate the likely costs of expanding the public sector workforce in line with the HR Strategy 2014–2018. In an existing draft policy spanning the period 2019–2023, health workforce expansion is estimated to cost Ksh 79 billion (around £600 million) of which Ksh 531.0 million (around £4 million) will be used to train all postgraduate specialists in each year.^13^

However, the authors and their key contacts are unaware of any specific funding or planning provisions for expanding the paediatric specialist workforce in the public sector.

## Discussion

The paediatric population of Kenya is approximately 25 million young adolescents, children and newborns who require healthcare.^9^ The burden of disease in this population is still primarily due to preventable communicable diseases, with an increasingly recognised burden of non-communicable diseases, violence, injuries and mental health conditions.^19^ There is continued need for skilled leadership in traditional areas of preventive and promotive child health such as nutrition, immunisation and early child development and in care of serious acute paediatric and neonatal conditions. However, greater attention also now needs to be paid to the ability of health systems to respond to the more complex needs of older children and adolescents to deliver on the survive, thrive and transform agenda.^20^ With Kenya and Africa’s population growing rapidly, the need to develop a context-appropriate skilled workforce is acute. It is not clear, however, that there is any comprehensive strategy to address this need.

Over 30% of WHO Member States report to have <100 medical doctors per 100 000 population.^21^ Countries with a higher burden of disease have fewer healthcare workers. The area most in need is sub-Saharan Africa which has 22% of the global burden of disease, access to only 3% of healthcare workers and <1% of the world’s financial resources.^21^ Kenya has only 19 medical doctors per 100 000 population, among the lowest fifth of all countries in the world, a ratio twice and four times lower than Nigeria and India, respectively.^13^ It has licensed paediatricians and public sector county paediatricians ratios of 1.33 and 0.41 per 100 000 of the population aged <19 years. The Ministry of Health’s ‘Reproductive, Maternal, Newborn, Child and Adolescent Health Investment Framework 2016–2030’ recognises these gaps and aims to improve service coverage at all levels of the health system based on the WHO global strategy.^22^ It does not, however, provide the strategy and financing detail for training appropriate numbers or categories of health workers to deliver these policy goals.

In consequence, the situation now, and one that will likely persist for many years, is that most inpatient paediatric and neonatal care in Kenyan county government hospitals is provided by prelicensure or ‘intern’ medical and non-physician clinician graduates (the latter have 3 years diploma training), who are under the supervision of junior general medical officers.^23^ Walk-in paediatric outpatient care in county hospitals and all smaller public facilities is typically provided by clinical officers and nurses. In the public sector, therefore, the limited numbers of paediatricians and their resource-poor environments means their major practical role is oversight of care initiated, continued and provided by more junior professionals. To complement the roles of specialist paediatricians, the Health Facility Assessment Survey conducted in 2018 identified only 255 clinical officers and 119 nurses nationally with higher diploma or master’s level paediatric qualifications, respectively while there remain very few family medicine specialists in Kenya.^11^

In contrast to this reality, the major focus of training for paediatricians is on gaining extensive and contemporary knowledge on the diagnosis and management of the full panoply of paediatric conditions, including those they may never be able to identify or treat in most parts of the public sector (eg, metabolic disorders). Meanwhile, training for needed clinical leadership, management, supervision, communication and quality improvement roles is very limited. This dissonance between the paediatric role as taught and imagined and its reality in county level facilities may be a major contributor to dissatisfaction and lack of engagement in dealing with the very real challenges of public sector paediatrics and child health.^24–26^

We therefore believe there is an urgent need for a process of consultation with all relevant stakeholders and the paediatric community to radically review thinking on who should provide public sector neonatal, child and adolescent health services at county levels in Kenya. This should include thinking about how paediatricians might work with a wider multiprofessional team that considers innovative options for task-sharing, an area of active policy development in Kenya.^26–28^ This may include greater attention paid to the development of clinical officers, nurses and even healthcare assistants with specific neonatal, paediatric and adolescent health skills to produce the most cost-effective skill-mix options to strengthen systems of paediatric care.^29^ It may require a reimagining of the role of county paediatricians as abilities to plan, monitor, manage and lead multidisciplinary teams may become key skills. Such strategic shifts may require a major revision or diversification of existing postgraduate training curricula and continuous professional development strategies. This may appear to threaten existing institutions such as universities and the professional associations. Thus, it is essential to involve all stakeholders in this process and learn lessons from other settings facing similar challenges. For example, there are clearly tensions between meeting the needs of populations and counties for essential basic services and developing further subspecialist forms of training such as in cardiology or intensive care. In some countries, paediatricians may decide, for example, that paediatric primary and first-level hospital care should be managed by those trained in family medicine together with more specialist nurses and clinical officers. The paediatric community may then limit itself to regional or tertiary care roles and expanding specialty referral and outreach responsibility. Such long-term strategic choices will likely be influenced by the current stage of development of different professions in different countries.

Encouragingly, Kenya is a partner in the relatively new East, Central and Southern African, College of Paediatrics and Child Health. The development of a College should enable standardisation and improvement in the quality of training, will allow for new developments in the curriculum such as a greater focus on paediatric primary care and aims to decentralise training to accredited sites in county hospitals (Thomas Ngwiri, personal communication, 2019). This could improve equity of access to paediatricians, understanding of their future leadership role in counties and promote career development opportunities for those not pursuing further subspecialty training. This could facilitate retention of professionals at county level and expand local training capacity for multiple cadres.

The development of a college must be considered alongside the continued roles of universities and schools that train other cadres as part of a long-term workforce strategy that encompasses issues of financing, recruitment and retention.^30 31^ Kenya is graduating more doctors who might become the paediatricians of the future, but this has not been accompanied by an increase in funding for their initial recruitment. There are worrying parallels with developments in the nursing profession where despite training relatively large numbers Kenya has a critical shortage of nurses in the public sector as a direct consequence of inadequate financing.^32^ Furthermore, public sector health workers often face delays in payment and an erosion of working standards that has contributed to widespread health worker strikes.^33^ Inadequate financing and poor human resource management must also therefore be addressed in the short term and governments must work towards meeting the Abuja targets for financing health after 10 years of relative inaction.

The limited availability of workforce information in forms that can be readily analysed means our findings should be interpreted with caution. Our focus was largely on the public sector and we do not attempt to make any specific comment on the contribution of the private or faith-based sectors. The former are an increasingly important source of care in major cities and towns to higher socioeconomic groups. Some faith-based providers make an important contribution in rural and some more disadvantaged urban regions, including some subspecialist care. Despite these limitations, we feel our findings raise important issues on the future provision of paediatric services for Kenya and similar LMIC, and thus for achieving UHC and the SDGs.

## Conclusion

A very small number of paediatricians is available to care for many millions of those aged <19 years in Kenya, especially those from lower income households. The scale of the workforce challenge in addressing gaps in neonatal, child and adolescent healthcare requires innovative solutions. Professional paediatric communities in LMIC like Kenya could play a leadership role in developing solutions through strategies that make the best use of nursing, non-physician and other physician cadres to best meet population and system needs.
