# Supplementary material for: The paediatrician workforce and its role in addressing neonatal, child and adolescent healthcare in Kenya
Source: Arch Dis Child. 2020 Jun 18;105(10):927–31. doi: 10.1136/archdischild-2019-318434 (PMC7513261; doi:10.1136/archdischild-2019-318434)
Supplement: Supplementary data [file archdischild-2019-318434supp001.pdf]

List of Kenya policy documents reviewed for the paper: *The paediatric workforce and its role in addressing neonatal, child and adolescent health care in Kenya*

Kenya Gazette. Supplement 195. 14 December 2012. The Medical Practitioners and Dentists Act. Fourth Schedule Part 1. Specialties in Medical Practice. Part 11. Sub-Specialties in Medical Practice. 2012.	Supplementary source
Kenya Medical Practitioners and Dentists Board. Approved Institutions for MBChB and BDS Training. Website accessed 21.3.2019.	Referenced (18)
Kenya National Health Accounts. FY 2015/2016.	Supplementary source
Kenya Paediatric Association. Strategic Plan. 2018-2022.	Supplementary source
Kenya Ministry of Health. Human Resources for Health Norms and Standards Guidelines for the Health Sector. 2014.	Referenced (15)
Kenya Ministry of Health. Kenya Human Resources For Health Strategic Plan (KHRHSP). 2019 – 2023 (Final draft).	Referenced (13)
Kenya Ministry of Health. Health Infrastructure Norms and Standards. 2017.	Supplementary source
Kenya Ministry of Health. Health Sector Human Resources Strategy 2014-2018. 2014.	Referenced (12)
Kenya Ministry of Health. Kenya Harmonised Health Facility Assessment Survey. 2018.	Referenced (11)
Kenya Ministry of Health. Kenya Health Sector Strategic and Investment Plan 2014 – 2018. 2014.	Referenced (17)
Kenya Ministry of Health. Kenya Health Policy 2014 – 2030. 2014.	Supplementary source
Kenya Ministry of Health. Kenya Health Workforce Report: The status of healthcare professionals in Kenya, 2015. Website dated 2017.	Referenced (16)
Kenya Ministry of Health. Report of the Training Needs Assessment of Kenya's Health Workforce, 2015.	Supplementary source
Kenya Ministry of Health. Reproductive, Maternal, Newborn, Child and Adolescent Health Investment Framework 2016-2030. 2016.	Referenced (22)
Kenya Ministry of Health. Statistical Review of Progress Towards the Mid-Term Targets KHSSP 2014-2018. 2016.	Referenced (19)
Kenya Ministry of Health. Task Sharing Policy Guidelines. 2017–2030. 2017.	Supplementary source
Kenya Ministry of Health. Policy Brief. Masibo R, Kiarie H and Bartilol P. Human Resources for Health: Gaps and Opportunities for Strengthening. 2019.	Supplementary source
Kenya Ministry of Health. Wangai E and Charles K. Policy Brief. Refocusing on quality of care and increasing demand for services: Essential elements in attaining universal health coverage in Kenya. 2019. http://www.health.go.ke/wp-content/uploads/2019/01/UHC-QI-Policy-Brief.pdf	Supplementary source
Ministry of Public Health and Sanitation. Ministry Medical Services. National HRH Strategic Plan 2009 – 2012. April 2009.	Supplementary source

Republic of Kenya. Health Bill. 2015.	Supplementary source

List of World Health Organisation policy documents reviewed for the paper: *The paediatric workforce and its role in addressing neonatal, child and adolescent health care in Kenya*

World Health Organization and Global Health Workforce Alliance. Campbell J, Dussault G, Buchan J, Pozo-Martin F, Guerra Arias M, Leone C et al. A universal truth: no health without a workforce. Forum report, Third Global Forum on Human Resources for Health, Recife, Brazil. Geneva. 2013	Supplementary source
World Health Organization and Global Health Workforce Alliance. Wafula,F. et al. Primary Health Care Systems (PRIMASYS) A case study from Kenya. 2017.	Supplementary source
World Health Organisation. Every Women Every Child: The global strategy for women's, children's and adolescents' health 2016-2030. 2016.	Referenced (4)
World Health Organisation. Health workforce requirements for universal health coverage and the sustainable development goals. Human Resources for Health Observer Series No 17. 2016.	Supplementary source
World Health Organisation. Models and tools for health workforce planning and projections. Human Resources for Health Observer. NO 3. 2010.	Referenced (30)
World Health Organisation. Global Strategy on Human Resources for Health: Workforce 2030. 2016.	Referenced (2)
World Health Organisation. Workload Indicators of Staffing Need. User's Manual. 2010.	Referenced (14)

List of commentaries reviewed for the paper: *The paediatric workforce and its role in addressing neonatal, child and adolescent health care in Kenya* – Supplementary sources

World Health Organisation. Kiambati,H. Kiio, C. Toweett J. Understanding the labour market of HRH in Kenya. Working Paper November, 2013.

Njenga,M. Position Paper No 1. Review of Human Resources for Health Policies in Kenya Policy Gaps and Agenda for the Reform. HRH Advocacy Project, World Vision, Kenya. 2015.

Position Paper No 5. Human Resources for Health Issues in Kenya. Constraints and opportunities from a recent baseline survey. HRH Advocacy Project, World Vision, Kenya. 2015.

Taddese A.& Lehmann J. Human Resources for Health in Kenya: A stock and flow review. FUNZAKenya and IntraHealth International. 2017.
